# Alterations in the glycome after HDAC inhibition impact oncogenic potential in epigenetically plastic SW13 cells

**DOI:** 10.1186/s12885-018-5129-4

**Published:** 2019-01-16

**Authors:** McKale R. Montgomery, Elizabeth E. Hull

**Affiliations:** 1 College of Human Sciences, Oklahoma State University, Stillwater, OK 74078 USA; 2 Biomedical Sciences Program, College of Graduate Studies, Midwestern University, Glendale, AZ 85308 USA

**Keywords:** Glycome expression, HDAC inhibition, Chemoresistance, Gene expression profiling, Glycan Array, Lectin Array, Epigenetics

## Abstract

**Background:**

Defects in the type and degree of cellular glycosylation impact oncogenesis on multiple levels. Although the type of glycosylation is determined by protein sequence encoded by the genome, the extent and modifications of glycosylation depends on the activity of biosynthetic enzymes and recent data suggests that the glycome is also subject to epigenetic regulation. This study focuses on the ability of HDAC inhibition to alter glycosylation and to lead to pro-oncogenic alterations in the glycome as assessed by metastatic potential and chemoresistance.

**Methods:**

Epigenetically plastic SW13 adrenocortical carcinoma cells were treated with FK228, an HDAC inhibitor with high affinity for HDAC1 and, to a lesser extent, HDAC2. In comparing HDAC inhibitor treated and control cells, differential expression of glycome-related genes were assessed by microarray. Differential glycosylation was then assessed by lectin binding arrays and the ability of cellular proteins to bind to glycans was assessed by glycan binding arrays. Differential sensitivity to paclitaxel, proliferation, and MMP activity were also assessed.

**Results:**

Treatment with FK228 alters expression of enzymes in the biosynthetic pathways for a large number of glycome related genes including enzymes in all major glycosylation pathways and several glycan binding proteins. 84% of these differentially expressed glycome-related genes are linked to cancer, some as prognostic markers and others contributing basic oncogenic functions such as metastasis or chemoresistance. Glycan binding proteins also appear to be differentially expressed as protein extracts from treated and untreated cells show differential binding to glycan arrays. The impact of differential mRNA expression of glycosylation enzymes was documented by differential lectin binding. However, the assessment of changes in the glycome is complicated by the fact that detection of differential glycosylation through lectin binding is dependent on the methods used to prepare samples as protein-rich lysates show different binding than fixed cells in several cases. Paralleling the alterations in the glycome, treatment of SW13 cells with FK228 increases metastatic potential and reduces sensitivity to paclitaxel.

**Conclusions:**

The glycome is substantially altered by HDAC inhibition and these changes may have far-reaching impacts on oncogenesis.

**Electronic supplementary material:**

The online version of this article (10.1186/s12885-018-5129-4) contains supplementary material, which is available to authorized users.

## Background

Proper glycosylation of the dense layer of glycoconjugates on the cell surface is necessary for effective communication between cells and the environment. Aberrant glycosylation in cancer is associated with disrupted cell signaling, evasion of growth suppressors, immunoresistance, increased angiogenesis, and induction of invasion and metastasis [1–3]. Furthermore, both defective and increased levels of glycosylation have been shown to contribute to multidrug resistance in various cancers [4, 5]. As such, a better understanding of the cancer glycome and glycoproteome will be instrumental to the identification of more definitive cancer biomarkers and development of more effective cancer therapeutics.

Ultimately, the versatility of glycan structures help cancer cells respond, adapt, migrate, and invade. Although there is no genetic template for glycan structure, the type of glycosylation added to any particular protein is unequivocally determined by consensus sequences in the gene encoding for it. The structure of the glycan added then depends on expression of the enzymes required to synthesize the carbohydrate glycan structures. Thus, glycome regulation is complex, and involves more than 600 so-called “glycogenes” which encode for the various proteoglycans, glycosyltransferases, glycosidases, sulfatases, and carbohydrate biosynthetic enzymes that determine glycome function. Evidence suggests that individual glycome composition is largely genetically predetermined, but recent advances in glycobiology have identified disease- and cancer-specific glycophenotypes which are under epigenetic control [6–8]. This is intriguing because unlike genetic mutations, epigenetic modifications are transient and reversible, making them extremely desirable therapeutic targets.

HDAC inhibitors are powerful epigenetic regulators and promising cancer therapeutics, but in solid tumors, these drugs are not effective as single agents, and instead are often use in combination with a chemotherapeutic. Furthermore, the broad-spectrum and often pleiotropic effects off HDAC inhibitors make it difficult to harness their full potential. Indeed, while growth rate is slowed by HDAC inhibitor treatment in nearly all cancer cells, many also increase oncogenic properties such as increased motility and invasive capacity [9]. Although other epigenetic regulators, such as the DNA methyltransferase inhibitor 5-Aza-dc, have been used extensively in multiple cancer types to effectively target glycogenes and glycosylation enzymes to slow tumor progression [10–12], the effects of HDAC inhibitors on glycosylation, oncogenic phenotype, and chemotherapeutic response have not been extensively investigated. At a broader level, even though epigenetic regulation determines many aspects of oncogenesis and normal cellular behavior, these epigenetic processes and their regulation are not well understood.

In the study of epigenetic regulation of the glycome, the human adrenal cortical carcinoma SW13 cell line represents an invaluable research model as it exists as two distinct phenotypes which are epigenetically plastic [13, 14]. In this study, we utilize the SW13 cell line as a model of epigenetic phenotype regulation to investigate how HDAC inhibitors can influence the acquisition of distinct oncogenic characteristics, such as rapid proliferation and metastatic capacity. Data presented here suggest that HDAC inhibition significantly impacts the SW13 glycome. Specifically, we demonstrate differential glycogene expression in multiple biosynthetic pathways and document changes in glycome composition. We then go on to characterize the glycosylation signatures and glycan binding preferences of tumorigenic versus metastatic SW13 cells. Finally, we demonstrate that the changes induced by HDAC inhibitor treatment observed in the SW13 glycome are associated with decreased chemosensitivity. This work furthers our understanding of how HDAC inhibition might influence malignant transformation in cancer cell types that respond negatively to HDAC inhibitor treatment. This study also identifies candidate biomarkers for determining responsiveness to HDAC inhibition and chemotherapeutic treatments.

## Methods

### SW13 cell culture and treatment

SW13 cells were obtained from American Type Culture Collection (ATCC; CCL-105) and maintained in high glucose DMEM supplemented with 10% fetal bovine serum and 10 U/ml penicillin/streptomycin at 37 °C in a humidity controlled incubator. For HDAC inhibition, cells were treated with 1 nM romidepsin (FK228) (Selleckchem) for 24 h.

### Morphology, proliferation, and MMP activity assays

Morphology was assessed via immunofluorescence using standard techniques. Specifically, SW13 control cells, or SW13 cells that had been treated with 1 nM FK228 were fixed with 4% paraformaldehyde, permeabilized with 0.2% Triton-X, and blocked with 1% BSA before a 1 h incubation with 25 U/ml Alexafluor 488 phalloidin (Invitrogen). Samples were mounted with ProLong Gold anti-fade reagent with DAPI to stain nuclei and photographed using a Zeiss Axiovert apotome with a 40X objective lens and a uniform exposure at each wavelength.

SW13 cell proliferation with and without 1 nM FK228 treatment was measured using a Click-iT EdU assay (ThermoFisher) according to the manufacturer’s instructions. Cells were imaged using a Zeiss Axiovert Apotome with a 10X objective lens and uniform exposure at each wavelength. Percent proliferation was quantified using NIH ImageJ software to divide the number of cells which stained positive for EdU by the total number of cells in each groups.

MMP activity was measured by plating SW13 cells at 1 × 10^4^ cells/well in 8-chamber slides. After 24 h of no treatment (control) or treatment with 1 nM FK228 cells were incubated overnight with a DQ gel substrate (Life Technologies) diluted to 40 μg/ml in MMP activity buffer (100 mM NaCl, 100 mM Tris-HCl, pH 7.5, 10 mM CaCl_2_, 20 μM ZnCl, 0.05% NP40, 0.2 mM sodium azide). Cells were then washed 2 times with 1X PBS before fixation and DAPI staining. Relative differences in MMP activity were quantified using NIH ImageJ software to divide the relative amount of green fluorescence by the number of cells in a given well. All experiments were performed at least in triplicate.

### Gene expression analysis of SW13 cells following HDAC inhibitor treatment

Total RNA was isolated from cells using Trizol according to the manufacturer’s recommendations. Following quality control analysis (260/230 > 1.5, 260/280 > 1.8, RIN > 6), whole genome microarray analysis was performed by PhalanxBiotech using their OneArray platform. Standard selection criteria to identify differentially expressed genes were established as log2 fold change ≥1 and *p* < 0.05. For advanced data and pathway analysis, intensity data were pooled and calculated to identify differentially expressed genes based on the threshold of fold change and *p*-value. A gene set enrichment analysis of Gene Ontology (GO) terms was then performed using the differentially expressed gene lists as input. A small subset of differentially expressed genes identified in the array were selected for validation by qRT-PCR. Briefly, the same total RNA described above was reverse transcribed using SuperScript II, and qRT-PCR was performed using 50 ng cDNA per reaction on an ABI StepOne Plus thermocycler using SYBR Green chemistry. Expression changes were analyzed via the ^ΔΔ^C_T_ Method following normalization to GAPDH expression, which was utilized as an invariant control.

### Lectin and glycan array analysis

Lectin and glycan arrays offer a high-throughput approach for cellular glycoprofiling and the identification of preferential carbohydrate binding moieties, respectively. Thus, to assess the functional consequences of glycogene expression changes in response to HDAC inhibition, total protein from SW13 control cells and SW13 cells that had been treated with 1 nM FK224 for 24 h were subjected to lectin and glycan array analysis (*n* = 4 per group). Arrays were performed and analyzed by RayBiotech. The Lectin 40 array is designed to detect glycan profiles using 40 unique lectins, while the Glycan 100 array assess carbohydrate binding preferences using the 100 most frequently identified structures showing important binding function in the literature. Lists of all lectins and glycans utilized in the arrays are available in Additional file 1: Tables S1 and S2.

### Lectin binding analysis of carbohydrate expression

Binding of select lectins were validated by lectin blot analysis wherein 25 μg total protein from SW13 control or FK228 treated cells was electrophoresed through a pre-cast 4–20% polyacrylamide gel and then transferred to a PVDF membrane. Membranes were blocked with Pierce SuperBlock® buffer for 30 min, and then incubated with 20 μg/ml FITC labeled lectins (EY Laboratories) for ~ 2 h before being imaged using a ChemiDoc. Alternatively, treated and untreated cells were fixed as described above before incubation with 20 μg/ml FITC labeled lectins for ~ 2 h. Samples were mounted with ProLong Gold anti-fade reagent with DAPI to stain nuclei and photographed using a Zeiss Axiovert apotome with a 40X objective lens and a uniform exposure at each wavelength. To demonstrate specificity of lectin binding, wheat germ agglutinin (WGA) stained cells were incubated with WGA elution buffer for 30 min for mounting and visualization.

### Measuring GAG-sulfation

Total GAG-sulfation was assayed using a 1,9-Dimethylmethylene blue (DMB) assay as previously described [15]. DMB is a cationic dye that specifically binds to sulfated glycosaminoglycans with an absorbance at ~ 525 nm. Briefly, ~ 1 × 10^6^ cells were collected in 40 μl PBS or RIPA buffer. For the cells in PBS, 125 μl DMB buffer (31 μM DMB in 55 nM formic acid, pH 3.5) was added and transferred to a clear-walled 96-well plate. DMB binding was quantified by measuring the optical density at 525 nm. Total protein levels were assessed by BCA assay from the cells in RIPA buffer. DMB binding levels were normalized to total protein levels.

### Viability assays

Chemotherapeutic sensitivity was measured using an MTT (3-(4,5-Dimethylthiazol-2-yl)-2,5-diphenyltetrazolium bromide) assay. Cells were plated at 3 × 10^4^ cells per well in 96-well plates and incubated with 1, 10, or 50 nM paclitaxel (Cayman Chemical) for 24 or 48 h. Following treatment, 10 μl of 12 mM MTT was added to each well and cells were placed back in the incubator at 37 °C for 4 h. The formazan was solubilized by adding 100 ul solubilization solution (40% DMSO, 16% SDS, 2% acetic acid) followed by another incubation at 37 °C for 1 h. Spectrometric absorbance at 570 nm was measured with a microplate reader. Each assay included four technical replicates and was repeated at least three times. Paclitaxel effects at each dose, and differences in paclitaxel response between cell lines were analyzed using a two factor mixed design ANOVA using SPSS version 19 software (Chicago, IL). A Bonferroni test was used to adjust for multiple pairwise comparisons. Mean differences were considered statistically significant at *p* < 0.05.

## Results

### HDAC inhibition induces morphologic and phenotypic changes in SW13 cells

**Fig. 1:**
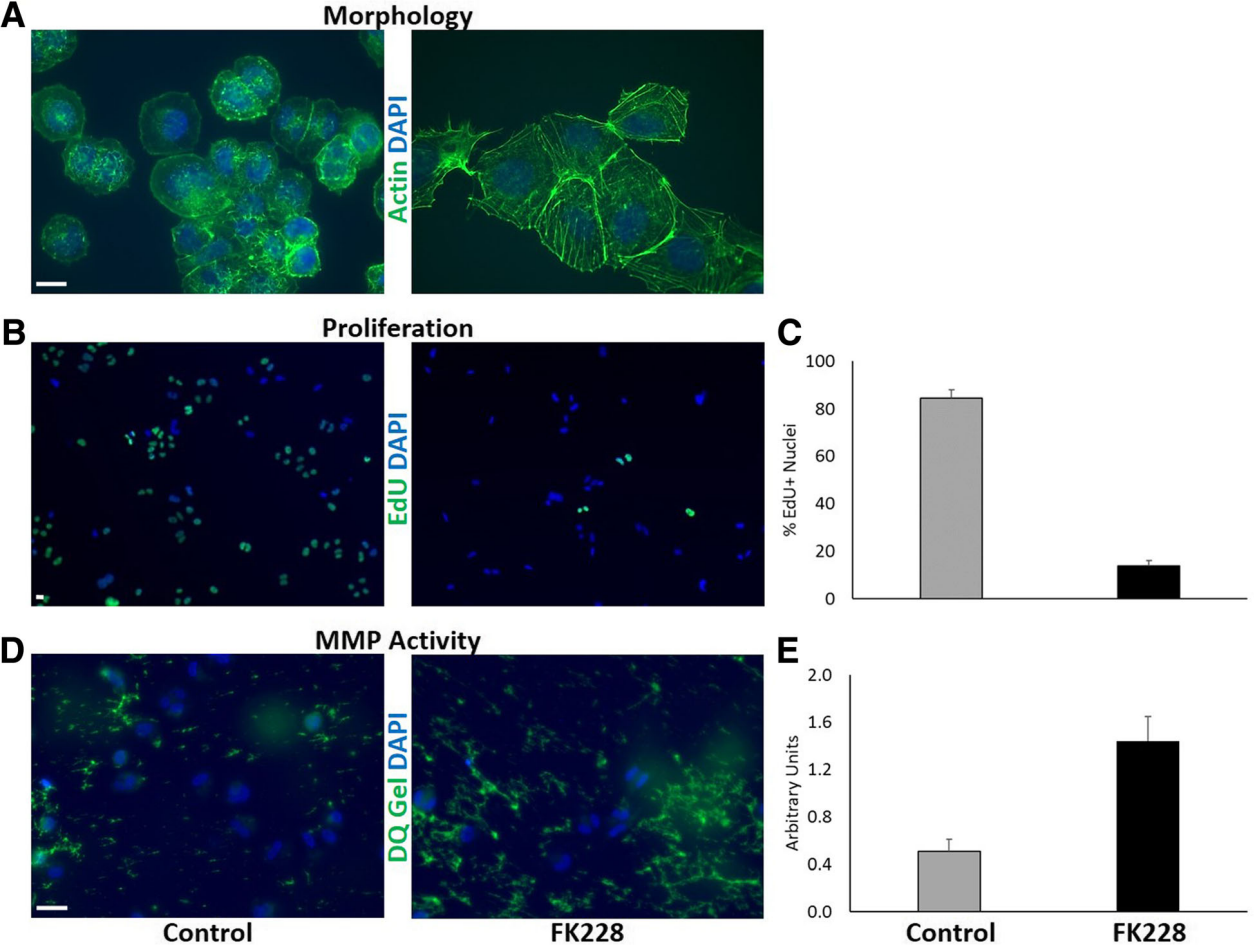
HDAC inhibition induces changes in SW13 morphology, proliferation, and MMP activity. (A) Treatment with 1 nM FK228 significantly impacts SW13 cell morphology and actin (green) organization. (B and C) FK228 treatment decreases proliferation by nearly 90% as determined by the lower number of EdU positive cells (green) per total number of cells indicated by DAPI staining (blue). (D and E) FK228 treated cells exhibit higher levels of MMP2/9 (collagenase) activity as measured by increased fluorescence after incubation with a fluorescein-quenched gelatin substrate (DQ gel; green). Scale bars represent 10 μm

Previous work has demonstrated that the SW13 cell line can exist at two distinct subtypes, and that subtype determination is epigenetically regulated [13, 14, 16]. One subtype (SW13-) is a rapidly growing, highly tumorigenic, epithelial-like subtype, while this other (SW13+) is a slow growing, metastatic, more mesenchymal-like subtype [14]. The two subtypes are typically distinguished by the presence (SW13+) or absence (SW13-) of the intermediate filament protein vimentin and the expression of the tumor suppressor protein BRM (a subunit of the SWI/SNF chromatin remodeling complex with ATPase activity). HDAC inhibitors have been shown to induce the expression of these proteins [14, 16]. To evaluate the capacity for HDAC inhibition to influence SW13 subtype phenotype and oncogenic characteristics, SW13 cells were treated with 1 nM FK228 for 24 h, after which morphology, proliferation, and MMP activity were assessed. Figure 1 a shows representative images of control SW13 cell morphology and SW13 cell morphology following treatment with FK228. Under control conditions, the majority of SW13 cells are small, rounded, epithelial cells that tend to grow very rapidly (SW13- phenotype) (Fig. 1 a). However, treatment with 1 nM FK228 significantly impacts SW13 cell morphology and actin organization (Fig. 1 a), and significantly slows SW13 cell proliferation (Fig. 1 b and c). FK228 treatment however also significantly increases SW13 cell collagenase activity (Fig. 1 d and e). Collectively, these results demonstrate that FK228 treatment induces a subtype switch from a rapidly growing, epithelial-like SW13- subtype to a slow growing, but more invasive, mesenchymal-like SW13+ subtype. Interestingly, this phenomenon is not limited to the SW13 cell line. Lin et al. showed similar responses of HDAC inhibitor-induced increased metastatic capacity in 13 other human cell lines, both in vitro and in vivo [9]. Because of the vast interest in using HDAC inhibitors as cancer therapeutics, we were interested in better understanding the mechanisms underlying this type of negative response.

### HDAC inhibitor treatment significantly impacts expression of SW13 glycome genes

As a first approach to identifying how HDAC inhibitor treatment can lead to such dramatic changes in oncogenic properties, we performed microarray analyses to assess global gene expression changes in the SW13 cell line following HDAC inhibition. We chose to continue with the SW13 cell line because of its two well defined epigenetic phenotypes, and its well characterized response to HDAC inhibition. First, SW13 cells were treated with 1 nM FK228 for 24 h, total RNA was isolated, and then submitted for microarray analysis. Following HDAC inhibition, the expression of 1760 genes was increased, while 1490 genes had decreased expression (Additional file 1: Table S3). A gene set enrichment analysis of Gene Ontology (GO) terms was then performed using the differentially expressed gene lists as input. Top enrichment GO terms based on biological processing included negative regulation of cell differentiation, as well as regulation of cell migration and cell proliferation (Additional file 1: Table S4), which were consistent with the phenotypic changes observed in Fig. 1.

Included in the list of differentially expressed genes are enzymes in key pathways affecting the biosynthesis and degradation of most classes of glycolipids and glycoproteins, including enzymes which modify sulfation levels of these molecules (Table 1). Several differentially expressed enzymes are initiators in their respective biosynthetic pathways suggesting that the flux through these pathways may be substantially altered. Affected pathways include EGF domain modification, GPI-anchor biosynthesis, muscin-type O-linked oligosaccharide synthesis, and the core tetrasaccharide linkers of O-linked GAG synthesis (heparan sulfate proteoglycan (HSPG), chondroitin sulfate, and dermatin sulfate). Furthermore, in the chondroitin sulfate and HSPG biosynthetic pathways, the enzymes involved in the initial elongation of the core tetrasaccharide linker are differentially expressed. As over 50% of all proteins are glycosylated, glycosylation is recognized as the predominant post-translational modification. Thus, shifts in the enzymes which catalyze these modifications may have wide-ranging effects.

**Table 1:** Differentially expressed enzymes and their function within the glycome. Enzymes affecting metabolism of glycolipids and glycoproteins whose expression levels change in response to HDAC inhibition are grouped according to their affected pathway and whether the protein product is associated with cancer (are in bold) or is the initial step in the biosynthetic pathway (are in italic). References documenting the gene product link to cancer indicated in parentheses

Biosynthetic & other metabolic processes		
Log2 Fold-change	Symbol	Gene Name
Multiple Pathways		
−1.26	**FUT10**	α-(1,3)-fucosyltransferase 10 [46, 47]
−1.26	**FUT11**	α-(1,3)-fucosyltransferase 11 [46, 48, 49]
−1.56	**B4GALT2**	UDP-Gal:β-GlcNAc β-(1,4)-galactosyltransferase 2 [50]
1.07	B3GAT2	β-(1,3)-glucuronyltransferase 2
Glycoprotein		
EGF Domain		
−1.04	**POGLUT1**	*Protein O-glucosyltransferase 1* [50–53]
1.30	**LFNG**	O-fucosylpeptide 3-β-GlcNAc transferase [50, 54]
N & O-Linked Pathways		
1.56	**B3GNT2**	N-acetyllactosaminide β-(1,3)-GlcNAc transferase 2 [50, 55]
Complex N-Linked Pathway		
−1.10	**ALG13**	UDP-GlcNAc transferase subunit [50]
−1.09	**ALG10**	α-1,2-glucosyltransferase [56]
5.16	**MAN1A1**	α-Mannosidase, class 1A, member 1 [8, 52]
1.63	**MGAT4A**	α-(1,3)-mannosyl-glycoprotein 4-β-N-acetylglucosaminyltransferase A [50, 56]
Complex O-linked Pathway		
−1.28	**GALNT14**	*Polypeptide GalNAc transferase 14* [8, 57, 58]
1.00	**GALNT6**	*Polypeptide GalNAc transferase 6* [8, 50]
−1.08	**GALNT7**	GalNAc transferase 7 [8, 50, 59, 60]
1.79	**GCNT1**	β-(1,3)-galactosyl-O-glycosyl-glycoprotein β-1,6-GlcNAc transferase [50, 61, 62]
O-linked GAG synthesis		
Core tetrasaccharide linker for HSPG, Chondroitin Sulfate, Dermatan sulfate		
2.85	**XYLT1**	*Xylosyltransferase I* [50, 63]
−1.36	B3GALT6	UDP-Gal:βGal β-(1,3)-Gal transferase polypeptide 6 (GALT2)
Chondroitin Sulfate		
1.85	CGAT1	*Chondroitin sulfate N-acetylgalactosaminyltransferase 1*
HSPGs		
1.10	**EXT1**	*Exostosin glycosyltransferase 1* [50]
−2.22	**NDST1**	N-deacetylase/N-sulfotransferase [50]
1.30	**GLCE**	Glucuronic acid epimerase [64, 65]
Glycolipid metabolism		
1.07	**KDEL1**	KDEL motif-containing protein 1 [50]
1.07	**KDEL2**	KDEL motif-containing protein 2 [50]
Sphingolipid & Gangliosides (lactosylceramide modification)		
1.57	**A4GALT**	α-(1,4)-galactosyltransferase [50]
1.46	**ST3GAL5**	ST3 β-galactoside α-(2,3)-sialyltransferase 5 [50]
2.80	ST8SIA1	ST8 (α-N-acetyl-neuraminide α-(2,8) sialyltransferase 1)
1.30	ST6GALNAC3	ST6 (α-N-acetyl-neuraminyl-2,3-β-galactosyl-1,3)
GPI Anchor synthesis		
1.10	**PIGH**	Phosphatidylinositol GlcNAc transferase subunit H [50]
−1.67	PIGW	Phosphatidylinositol-glycan biosynthesis class W protein
−1.21	**PIGO**	GPI ethanolamine phosphate transferase 3 [50]
−1.13	**PIGU**	Phosphatidylinositol glycan anchor biosynthesis class U protein [50]
Polysialic acid		
2.71	**ST6GAL2 / SIAT2**	ST6 β-galactosamide α-2,6-sialyltranferase 2
1.27	**ST8SIA4 / SIA8D**	ST8 α-N-acetyl-neuraminide α-2,8-sialyltransferase 4 [50]
Sulfation levels		
General enzymes		
1.11	**PAPSS1**	3′-phosphoadenosine 5′-phosphosulfate synthase 1 [50]
−1.09	**CHST10**	carbohydrate sulfotransferase 10 [50]
Sulfatases (HSPG)		
2.94	**SULF1**	Sulfatase 1 [66, 67]
1.11	**SULF2**	Sulfatase 2 [66–68]
Protein sulfotransferase		
1.00	**TPST2**	Tyrosylprotein sulfotransferase 2 [50]
Lipid sulfotransferases - sphingolipid/ceramide		
1.38	**GAL3ST1**	Galactose-3-O-sulfotransferase 1 [69, 70]
N&O linked sulfotransferases		
1.35	CHST8	Carbohydrate (N-acetylgalactosamine 4–0) sulfotransferase 8
−1.67	**CHST9**	Carbohydrate (N-acetylgalactosamine 4–0) sulfotransferase 9 [71–73]
Chondroitin / Dermatan sulfate		
1.25	**CHST11**	Carbohydrate (chondroitin 4) sulfotransferase 11 (C4ST-1) [50]
1.05	**CHST12**	Carbohydrate (chondroitin 4) sulfotransferase 12 [50]
− 1.42	CHST14	Carbohydrate (dermatan 4) sulfotransferase 14
2.58	**GAL3ST4**	Galactose-3-O-sulfotransferase 4 [50]
Catabolic enzymes		
Lysomal enzymes		
1.39	NEU1	Neuraminidase 1 (lysosomal sialidase)
2.80	**FUCA1**	Fucosidase, α-L- 1, tissue [52]
Glycoprotein Unibiquitin ligases (ERAD pathway)		
1.03	**FBXO2**	F-box only protein 2 [50]
−3.01	**FBXO6**	F-box only protein 6 [50]
−1.66	**FBXO17**	F-box only protein 17 [50]
Metabolic enzymes		
1.67	**GALM**	Galactose mutarotase [50]

Interestingly, 84% (43/51) of the differentially expressed genes identified in this study are involved in glycome biosynthesis and have been linked to cancer (Table 1, highlighted gene symbol entries). Some have been characterized as cancer biomarkers linked to prognosis using clinical data, while others have been shown to affect patterns of oncogenesis in laboratory studies and others to alter sensitivity to chemotherapeutics. This suggests that the observed changes in expression of genes coding for glycolipid and glycoprotein biosynthetic pathways may collectively result in alterations in the oncogenic potential of FK228 treated cells.

### Differential expression of HSPG genes and HSPG binding proteins

In analyzing the differentially expressed genes in Table 1, we noted that FK228 treatment altered the expression of enzymes involved in determining heparan sulfate (HS) chain length and composition. Indeed, more than half (5/9) of the enzymes in the HSPG biosynthetic pathway were differentially expressed: xylosyltransferase I (XYLT1) and UDP-Gal:betaGal beta 1,3-galactosyltransferase polypeptide 6 (B3GALT6), which are involved in the synthesis of the core tetrasaccharide linker and exostosin glycosyltransferase 1 (EXT1), N-deacetylase/N-sulfotransferase (NDST1) and Glucuronic acid epimerase (GLCE), which function in the elongation of HSPGs. In addition, differential expression of sulfatases SULF1 and SULF2 suggests that the sulfation of these moieties may be altered with FK228 treatment. Although HSPG function is dependent upon the presence or absence of HSPGs and HSPG binding partners, the vast majority of these interactions are dictated by the presence and composition of HS chains on the HSPG core proteins.

In addition to potential alterations in HS composition, we noted a shift in expression of a significant number of genes coding for HS modified proteins and HSPG binding partners (Table 2). HSPGs can be broken into 3 broad classes based on cellular localization and all classes of HSPGs were affected by HDAC inhibition: membrane (glypicans 2, 3, and 4 and syndecans 1, 2, and 4), extracellular matrix (ECM) (collagens type IX alpha 1 and XII alpha 1, and testican), and secretory (serglycin). We next examined the expression of HSPG binding proteins with FK228 treatment in the microarray data. Notably, the expression of 31 different HSPG binding proteins were impacted (24 increased and 7 decreased) by HDAC inhibition. These gene products fall into only a few categories with the most abundant (16 of 31) being related to growth factor or cytokine signaling. In addition, 4 are differentially expressed ECM genes, 3 are adhesive proteins (including REG4 which is a mannose binding lectin), and 4 are proteases or genes which affect protease activity. Collectively, these changes in gene expression might be expected to alter the cellular environment and these changes may further modify growth factor signaling. For example, a highly differentially expressed protein, SERPINE2, has been linked to invasiveness in pancreatic cancer [17, 18].

**Table 2:** Differentially expressed heparan sulfate proteoglycans and heparan sulfate binding proteins

Category	Gene Symbol	Gene Name	Foldchange
Heparan Sulfate Proteoglycans	COL12A1	collagen, type XII, alpha 1	3.09
	GPC4	glypican 4	2.85
	GPC3	glypican 3	2.78
	SRGN	serglycin	2.34
	SPOCK1	sparc/osteonectin, cwcv and kazal-like domains proteoglycan	2.10
	GPC2	glypican 2	1.96
	SDC4	syndecan 4	1.23
	SDC2	syndecan 2	1.22
	COL9A1	collagen, type IX, alpha 1	1.20
	SDC1	syndecan 1	−1.34
Heparan Sulfate Proteoglycan Binding Proteins	SERPINE2	serpin family E member 2	4.10
	BMP4	bone morphogenetic protein 4	3.95
	THBS1	thrombospondin 1	3.73
	ADAMTS15	ADAM metallopeptidase with thrombospondin type 1 motif, 15	3.58
	Laminin	laminin, beta 1	3.49
	LPL	lipoprotein lipase	3.44
	CCL2	chemokine (C-C motif)	3.35
	SFRP1	secreted frizzled-related protein 1	3.25
	LAMA1	laminin, alpha 1	3.16
	FGFR2	fibroblast growth factor receptor 2	2.92
	FSTL1	follistatin-like 1	2.62
	NAV2	neuron navigator 2	2.42
	PGF	placental growth factor	2.31
	CYR61	cysteine-rich, angiogenic inducer, 61	1.93
	ANOS1	anosmin 1	1.77
	FGF12	fibroblast growth factor 12	1.64
	REG4	regenerating islet-derived family, member 4	1.56
	HBEGF	heparin-binding EGF-like growth factor	1.44
	TGFBR3	transforming growth factor, beta receptor III	1.42
	WNT4	wingless-type MMTV integration site family, member 4	1.38
	FBN1	fibrillin 1	1.30
	LIPH	lipase, member H	1.16
	NCAM	neural cell adhesion molecule 1	1.15
	COL5A3	collagen, type V, alpha 3	1.05
	FGF10	fibroblast growth factor 10	−2.37
	HDGF	hepatoma-derived growth factor	−1.51
	FGF2	fibroblast growth factor 2 (basic)	−1.20
	ZNF146	zinc finger protein 146	−1.19
	AGER	advanced glycosylation end product-specific receptor	−1.18
	PCOLCE2	procollagen C-endopeptidase enhancer 2	−1.16
	PCSK6	proprotein convertase subtilisin/kexin type 6	−1.09

Within the microarray data, genes coding for multiple HSPGs and their binding partners are significantly differentially expressed after HDAC inhibitor treatment

### HDAC inhibition alters the expression of HSPGs and related genes

**Fig. 2:**
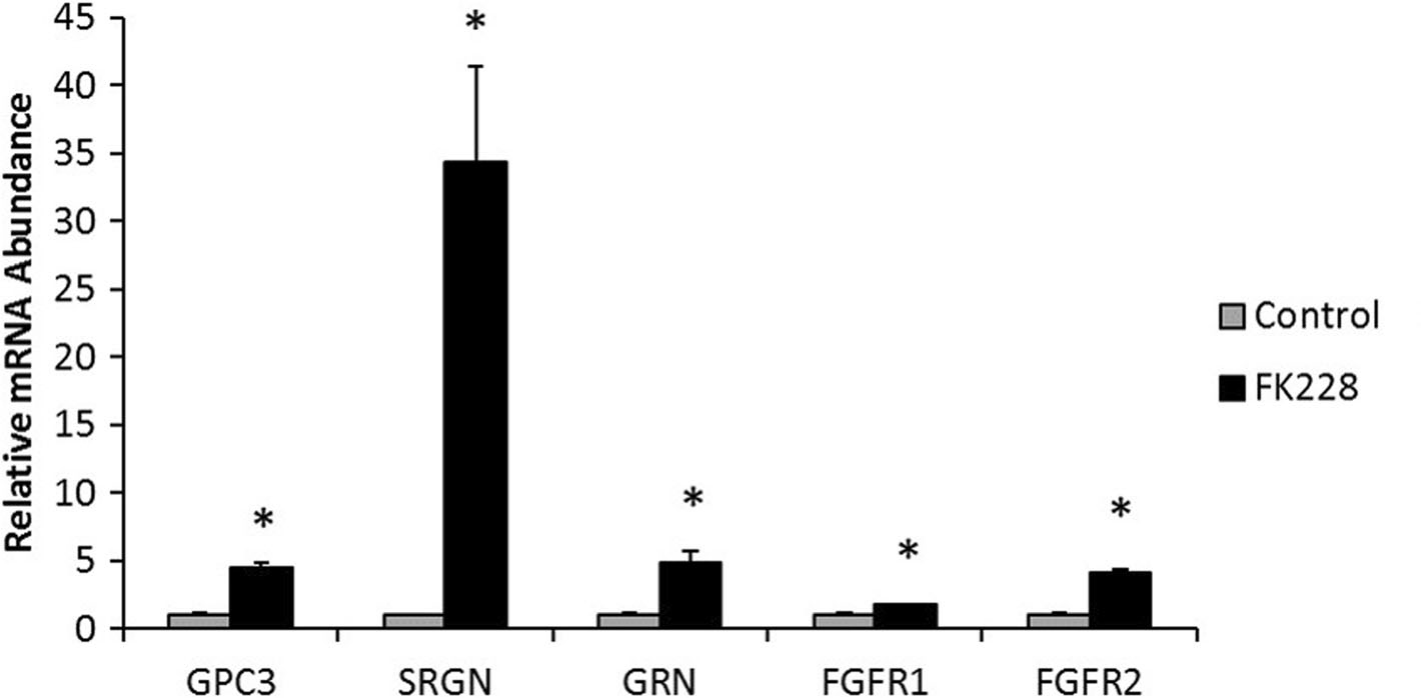
The expression of HSPGs and related growth factors are in HDAC inhibitor treated SW13 cells. Relative mRNA expression of glypican-3 (GPC3), serglycin (SRGN), granulin (GRN), and fibroblast growth factor receptor (FGFR) 1 and 2, in SW13 cells following treatment with 1 nM of the HDAC inhibitor FK228 for 24. *Denotes statistical difference from control, *p* < 0.05

Next, we quantitated expression of several genes to validate our array data by qPCR (Fig. 2). After treatment with FK228, expression of the HSGPs glypican-3 (GPC3) and serglycin (SRGN) increased by 5- and 35-fold respectively (Fig. 2), increases that are in the same direction as the gene chip data (which were 2.85 and 2.78 fold respectively). Although not HSPGs, FGFR1 and FGFR2 function as HSPG co-receptors. As shown in Fig. 2, mRNA levels for fibroblast growth factor receptors FGFR1 and FGFR2 are ~ 2- and 4-fold higher in FK228 treated cells as measured by qPCR. By comparison, in the microarray analysis expression of FGFR2 increases 2.92 fold while expression of FGFR1 does not change significantly. Granulin (GRN), a growth regulatory glycoprotein which promotes cancer progression and which interacts with HS [19], is differentially expressed as measured by qPCR by 5-fold.

### HDAC inhibition decreases cellular GAG-sulfation

**Fig. 3:**
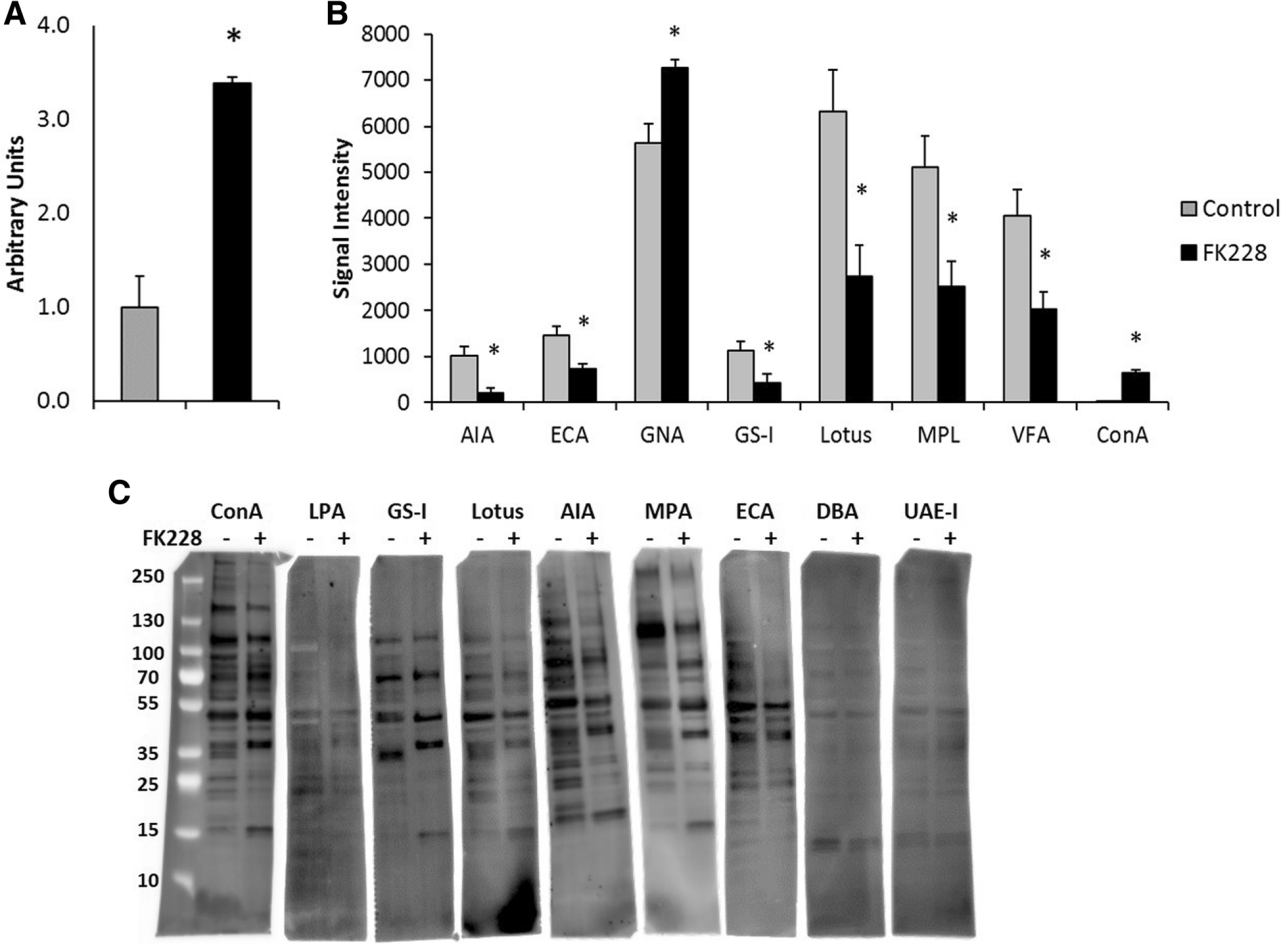
HDAC inhibitor treatment significantly impacts SW13 cell lectin binding and sulfation status. Total protein was isolated from SW13 control cells and SW13 cells that had been treated with 1 nM FK228 for 24 h (*n* = 4 per group). **a** FK228 treated SW13 cells have significantly higher levels of GAG-chain sulfation compared to SW13 control cells. **b** Lysates were subjected to lectin microarray analysis. Histograms of the fluorescence intensities lectins which were differentially bound between SW13 control cells and FK228 treated cells are shown. Data are presented as means ± SEM. **c** Lectin blots were performed to confirm lectin array results of select lectins (ConA, LPA, GS-I, Lotus, AIA, MPA, and ECA) that were differentially bound. DBA and UAE-1 were stained as negative controls. *Denotes statistical difference compared to control SW13- cells, *p* < 0.05.

As select genes for both HS modified proteins and their binding partners were differentially expressed, we next asked if the above gene expression changes resulted in detectable alterations in more global changes in the glycome. First, to assess whether the FK228 induced significant increases in the expression of many sulfotransferases had a functional impact on GAG-chain sulfation a DMB binding assay was performed. DMB binding was significantly higher in SW13 cells treated with 1 nM FK228 than in control SW13 cells (Fig. 3 a). These results indicate that total GAG-chain sulfation is significantly increased by HDAC inhibitor treatment. It should be noted that the DMB assay detects all changes in GAG sulfation and that this assay does not distinguish between changes in HSPG sulfation from the other classes of sulfated GAGs including chondroitin, dermatan, and keratan sulfate as well as hyaluronic acid. As sulfotransferases which modify chondroitin and dermatan are differentially expressed (CHST11, CHST12, CHST14), differential sulfation of these GAG structures may contribute to the results of the DMB assay. In addition, sulfation of non-GAG moieties may also be affected by HDAC inhibitor treatment as genes involved in modification of carbohydrates (CHST10), proteins (TPST2), lipids GAL3ST1), and N- and O-linked ST8, CHST9) are also differentially expressed.

### Lectin binding detects altered cellular glycan expression after HDAC inhibitor treatment

Based on the significant impact HDAC inhibition had on the expression of genes involved in GAG-chain sulfation and glycan biosynthesis, we hypothesized that FK228 treatment would also alter the glycosylation pattern of glycoproteins in treated SW13 cells. To test this hypothesis, total protein was isolated from SW13 control cells and SW13 cells treated with 1 nM FK228 for 24 h and assayed by lectin arrays. Array analysis revealed SW13 cells exhibit significant binding to several lectins when tested in a lectin array (fluorescent intensities ≥150 FU), with highest binding to *Vicia villosa* (VVA), *Hippeastrum* hybrid (HHA), and *Galanthus nivalis* (GNA), respectively, indicative of an abundance of GalNac and αMan containing glycoconjugates (Table 3). Though significant binding to lectins *Macckia amurensis* 1 (MAA), *Eruthrina cristagalli* (ECA), and *Griffonia Brandeiraea* (GS-II) suggests the presence of βGlcNac containing conjugates, 8 of the 17 lectins tested for which no binding was detected are also recognized by GlcNAc containing conjugates. This suggests an increased abundance of glycoconjugates with terminal βGlcNAc residues (Table 3 and Additional file 1: Table S2). FK228 treatment significantly increased binding to lectins Con A and GNA, while significantly decreasing binding to lectins *Artocarpus integrefolia* (AIA), *Erithrina crstagalli* (ECA), *Griffonia simplifcifolia I* (GS-I), *Lotus tetragonolobus* (Lotus), *Maclura pomifera* (MPL), and *Vicia faba* (VFA) (Fig. 3 b).

**Table 3:**
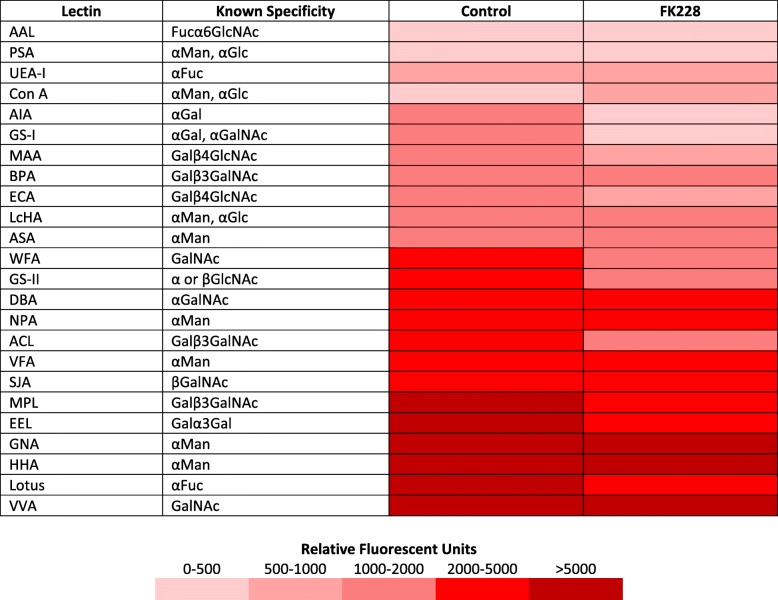
Comparison of lectin binding affinities between control and FK228 treated SW13 cells

Representative fluorescent signal intensities for binding to lectins of various glycan specificities of SW13 control cells and SW13 cells treated with 1 nM FK228 for 24 h. Fuc: L-Fuctose; Gal: D-Galactose; GalNac: N-Acetylgalactosamine; Glc: D-glucose; GlcNAc: N-Acetylglucosamine; Man: Mannose

**Table 4:**
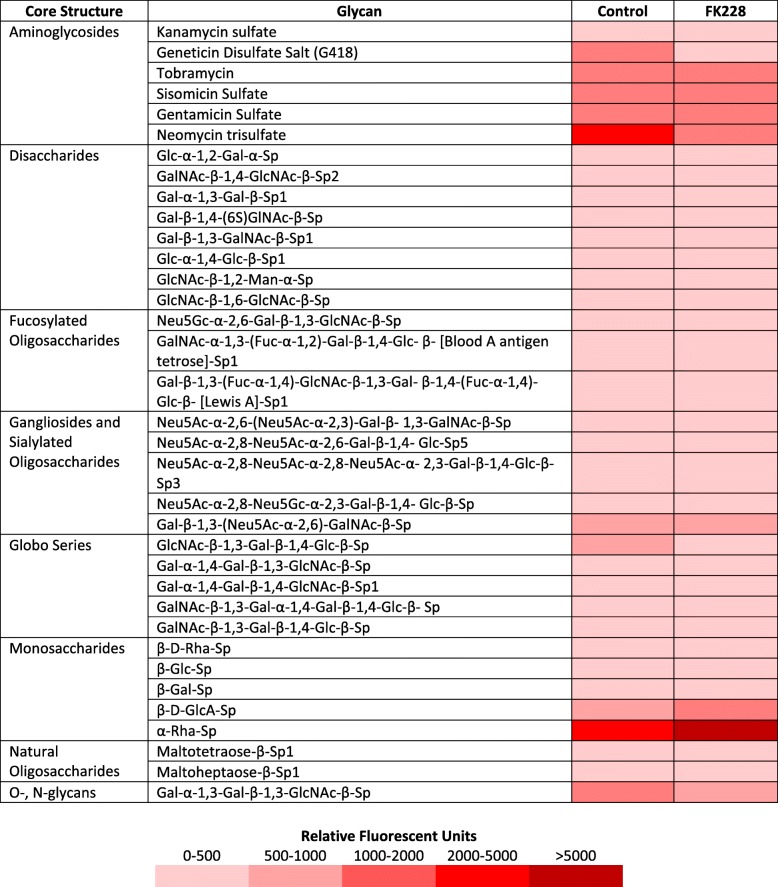
Comparison of glycan binding affinities between control and FK228 treated SW13 cells

Representative fluorescent signaling intensities summarizing the most intense glycan binding interactions for SW13 control cells and SW13 cells treated with 1 nM FK228 for 24 h. Fuc: L-Fuctose; Gal: D-Galactose; GalNac: N-Acetylgalactosamine; Glc: D-glucose; GlcNAc: N-Acetylglucosamine; Man: Mannose

Differential binding of select lectins was confirmed via lectin blot analysis (Fig. 3 c), an analysis which also assesses the molecular weights of glycosylated proteins. As seen in the array, FK228 treatment significantly increased the SW13 protein binding affinity for ConA, while it decreased the binding affinity for AIA, ECA, GS-I, Lotus and the profile of labeled proteins appears different after treatment (Fig. 3c). *Dolichos biflorus* (DBA) and *Ulex europaes I* (UAE-I) were included as negative controls, and also confirmed the lack of differential binding observed in the lectin array (Table 2 and Fig. 3 b). We noted that bands of different molecular weight were labeled with treatment in multiple lectin blots.

**Fig. 4:**
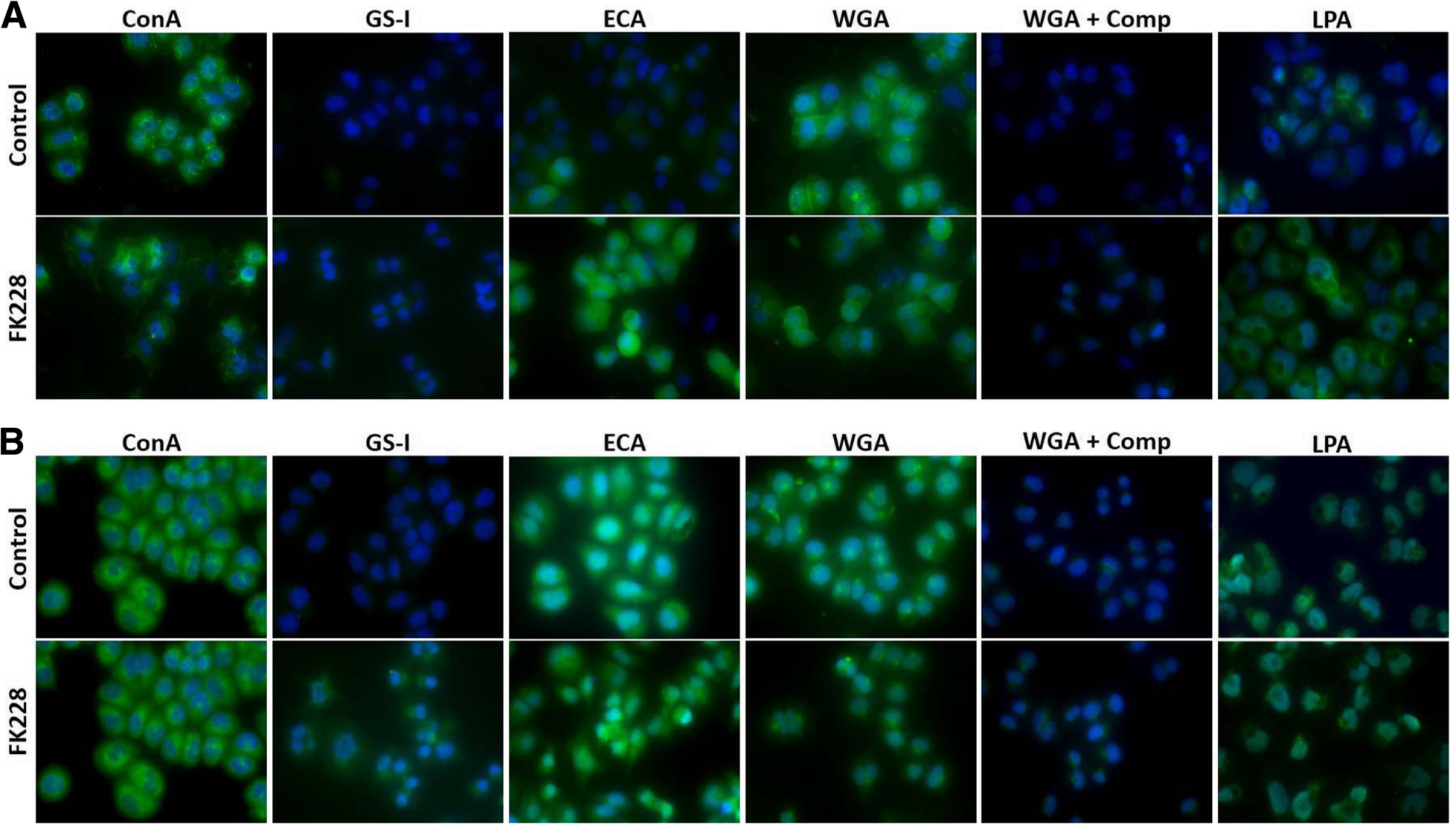
HDAC inhibition and sample preparation influence lectin binding in fixed cells. SW13 cells were left untreated (Control), or were treated with 1 nM FK228 for 24 h (FK228). Cells were then fixed and left unpermeabilized (**a**), or were permeabilized with 0.2% Triton-X (**b**) before incubation with 20 μg/ml FITC labeled lectins for ~ 2 h. To demonstrate specificity of lectin binding, wheat germ agglutinin (WGA) stained cells were incubated with WGA elution buffer (+ Comp) for 30 min. Permeabilization of fixed cells before incubation with FITC-labeled lectins can significantly impacts staining of some lectins. All samples were mounted with ProLong Gold anti-fade reagent with DAPI to stain nuclei and photographed using a Zeiss Axiovert apotome with a 40X objective lens and a uniform exposure at each wavelength

To expand on lectin array and blot data, we also analyzed lectin binding in fixed, cultured cells (Fig. 4). As many of the differentially expressed glycome genes are biosynthetic enzymes which act on both glycolipids and glycoproteins, we analyzed both permeabilized and unpermeabilized cultures. With detergent permeabilization, many glycolipids are extracted so that permeabilized cultures represent glycoprotein-rich samples while unpermeabilized cultures assess both glycolipids and glycoproteins. With this sample preparation, staining with ConA appears unchanged but GS-I and ECA labeling increase. These findings differ from the lectin array in relative intensities and direction of change. Interestingly, there was differential binding of WGA in the fixed samples. The WGA binding was effectively competed with competing sugar binding. We also assessed the binding of the sialic acid directed lectin LPA as genes related to sialic acid were differentially expressed (ST3GAL5, ST8SIA1, ST6GALNAC3, ST6GAL2, ST8SIA4, and NEU1). As shown in Fig. 4, normalized LPA binding increases 8.3-fold after 24 h of FK228 treatment in cells that have not been permeabilized (*p* = 0.033). However, this differential binding is not seen when cells are permeabilized before lectin binding. These data are supported by the observation that sialic acid is preferentially found in glycolipids [20].

### HDAC inhibitor treatment influences cellular expression of glycan binding proteins

Many of the biological effects of glycans are dependent upon their recognition by glycan binding proteins. Therefore, we next asked if the activity of glycan binding proteins was influenced by FK228 treatment. Of the 100 glycans tested in the array, significant binding (fluorescent intensities ≥150 FU) to 60 glycans was detected in SW13 control protein samples (Table 4, Additional file 1: Table S2). Treatment with 1 nM FK228 for 24 h significantly increased interactions of SW13 proteins with the aminoglycoside Neomycin trisulfate, and the monosaccharide α-Rha-Sp. However, FK228 treatment significantly decreased binding affinities for multiple other classes of glycans including two amino glycosides (geneticin disulfate and neomycin trisulfate), two disaccharides (Glc-α-1,2-Gal-α-Sp and GlcNAc-β-1,2-Man-α-Sp), one fucosylated oligosaccharide (GalNAc-α-1,3-(Fuc-α-1,2)-Gal-β-1,4-Glc- β-Sp1), three sialylated oligosaccharides (Neu5Ac-α-2,8-Neu5Ac-α-2,8-Neu5Ac-α- 2,3-Gal-β-1,4-Glc-β-Sp3, Neu5Ac-α-2,8-Neu5Gc-α-2,3-Gal-β-1,4- Glc-β-Sp, and Gal-β-1,3-(Neu5Ac-α-2,6)-GalNAc-β-Sp), and two natural oligosaccharides (Maltotetraose-β-Sp1 and Maltoheptaose-β-Sp1). Differences in binding interactions observed between SW13 control and FK228 treated samples are illustrated in Table 4.

### HDAC inhibitor pre-treatment promotes chemotherapeutic resistance

**Fig. 5:**
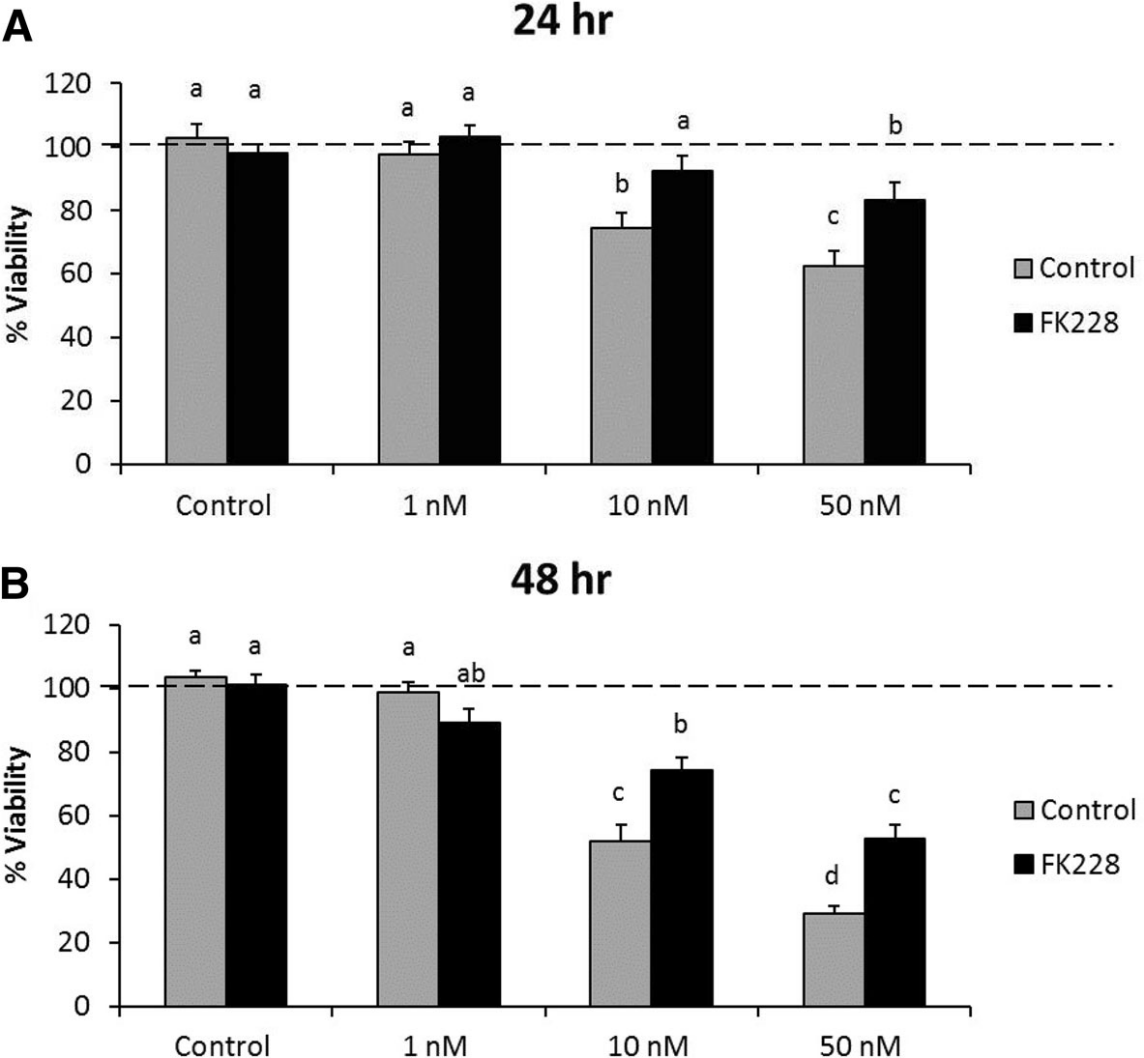
HDAC inhibitor treatment promotes paclitaxel resistance in SW13 cells. SW13 cells were left untreated (Control), or were treated with 1 nM FK228 for 24 h (FK228). After, 24 h, FK228 was removed, and control SW13 cells and FK228 pre-treated cells were plated into 96 well plates and left untreated (Control), or were treated with 1 nM, 10 nM, or 50 nM paclitaxel for (**a**) 24 h or (**b**) 48 h. Cell viability was assessed using an MTT assay. Data are presented as means ± SEM, and are representative of three independent experiments. Superscripts denote statistical significance, p < 0.05. Treatments that share the same superscripts are not significantly different

HDAC inhibitors are rarely effective as single agents, and are often used in combination with some other type of epigenetic agent or chemotherapeutic. However, changes in proteoglycan expression, glycosylation, and composition can also influence chemosensitivity and chemoresistance [7, 21, 22]. To assess how HDAC inhibitor treatment might impact the SW13 cell response to chemotherapeutic treatment, an MTT assay was used to evaluate the sensitivity of cells to paclitaxel treatment. Paclitaxel was chosen because SW13 cells are not sensitive to cisplatin or doxorubicin [23–25]. Cells were first cultured without treatment or with 1 nM FK228 for 24 h. After 24 h, both control and the FK228 treated cells were plated in fresh media containing no treatment (Control), or 1 nM, 10 nM, or 50 nM paclitaxel for 24 or 48 h. Neither the control SW13 cells nor the SW13 cells that had been treated with FK228 were affected by the 24 h 1 nM paclitaxel treatment (Fig. 5 a). However, the metabolic activity of control SW13 cells was significantly decreased compared to the FK228 pre-treated SW13 cells following 24 h of treatment with both 10 nM and 50 nM paclitaxel (Fig. 5 a). Similar patterns held true following 48 h of paclitaxel treatment, with no significant effect on metabolic activity in either the control or FK228 pre-treated SW13 cells at the 1 nM paclitaxel dose, but significant decreases in metabolic activity in both the control and FK228 treated cells at the 10 nM and 50 nM doses (Fig. 5 b). Of importance however, is that the metabolic activity of FK228 pre-treated cells was impacted significantly less than that of control cells in response to paclitaxel treatment at both the 10 nM (26% vs 49%) and 50 nM (48% vs 71%) doses (Fig. 5 b).

## Discussion

Data presented here suggests that HDAC inhibitor treatment of SW13 cells leads to > 2 fold changes in the expression of 3250 genes, a substantial portion of the genome. In our effort to characterize some of the additional functional consequences of HDAC inhibitor treatment, we documented significant impacts on SW13 cell morphology, growth, invasiveness, and chemotherapeutic response after HDAC inhibition. Importantly, these findings are not unique to the SW13 cell line, as numerous cell types have been shown to exhibit similar dramatic changes in gene expression and phenotypic characteristics with alterations in the glycome [9, 26, 27]. In addition, multiple HDAC inhibitors (including changes valporic acid, sodium butyrate, apicidin, MS-275, SAHA, and TSA) are able to illicit similar phenotypic changes in multiple cell lines [9]. Thus, we were confident SW13 cells and their response to FK228 treatment were a relevant model to investigate how HDAC inhibitor treatment contributes to such dichotomous epigenetically and phenotypically distinct oncogenic states.

Though in this study we used an HDAC inhibitor which primarily targets HDAC1, it is notable that multiple HDAC inhibitors have been demonstrated to lead to the characteristic phenotypic switch observed in the SW13 cell type [9, 13, 14, 16]. Thus, these phenotypic changes are likely not dependent on inhibition of a specific HDAC, but are likely mediated by widespread alterations in the acetylome that subsequently impacts global cellular gene expression. As inhibitors of lysine deacetylation however, HDAC inhibitor treatment affects the activity of many acetylated proteins, including many non-histone targets. Interestingly, only 3.9% of the acetylation sites detected after FK228 treatment of a colon cancer cell line were on core histones [28]. Recent work has demonstrated that acetylation patterns are tissue specific and have distinct subcellular distribution [29] and these patterns have been linked to changes metabolism and to regulate a wide variety of biological functions [28, 30, 31] and more importantly, acetylation of non-histone proteins has been linked to alterations in gene expression [32, 33]. Amongst the effects of alterations in the acetylome, we have chosen to address the effects of the glycome due to as recent data has suggested that the aberrant glycosylation seen in cancer is epigenetically regulated [34, 35].

Bioinformatics analyses of the microarray suggests that many glycome-related genes are differentially expressed after HDAC inhibitor treatment and many glycosylation pathways appear to be affected by treatment. Among the affected pathways, we found the prevalence of differentially expressed HSPG-related genes in the microarray data notable due to the link of these gene products with oncogenesis. Based on the data presented here, we suggest that HDAC inhibitor- mediated epigenetic regulation of the glycome, and possibly the HPSGs and their biosynthetic machinery, is an important contributory factor to the prevention and progression of oncogenesis.

Given the broad spectrum of differential expression of genes involved in glycome biosynthesis, alterations in lectin binding might be expected and lectin array analysis does indeed demonstrate HDAC inhibition significantly impacts the glycosylation signature of SW13 cell. We found it interesting that the profile of glycosylated proteins recognized by several lectins was also altered. This suggests alterations both in the amount and type of glycosylation, and that these glycan structures are attached to proteins of different molecular weights. Of particular interest is the increase in labeling by the sialic acid binding lectin LPA as increase in this glycosylation is associated with more aggressive tumors [26, 27]. In addition, the fact that this differential binding is not seen when cells are permeabilized underscores need to carefully consider sample preparation when comparing different lectin assays. Specifically, cellular glycosylation is most closely associated with membrane lipids and proteins and these glycans are susceptible to extraction with detergent permeabilization. In addition, it is clear that some cytosolic and nuclear proteins are glycosylated and can be bound by lectins (e.g. WGA and UEA) [36, 37] and these proteins may be enriched relative to other proteins due to sample preparation methods.

Importantly, changes in lectin binding corresponded with changes in mRNA expression identified in the microarray, and demonstrate the functional impact of HDAC inhibitor-mediated glycogene expression. For example, expression of the sialylatransferases ST6Gal2 and ST3GAL5 were significantly increased in HDAC inhibitor treated SW13 cells. Sialyltransferases function by linking sialic acids to terminal GalNAc and GlcNAc sugar residues on glycoproteins, and play important roles in cancer progression [38]. Decreased ECA and GS-II binding in HDAC inhibitor treated SW13 cells indicates increased masking of GlcNAc residues, consistent with increased sialyltransferase activity. In-line with our results here, this pattern of activity has also been associated with increased invasive capacity [27].

Similarly, decreased binding to ECA, as well ConA, has also been observed in mammary gland epithelial cells going through TGFβ induced epithelial to mesenchymal transition, which is also associated with cancer metastasis [39]. Moreover, these changes in lectin binding were also associated with similar changes in N-glycan-related gene expression changes observed in our study as well. Thus, it is interesting to note decreased binding to both of those lectins in response to HDAC inhibitor treatment. VFA binding was also decreased in the HDAC inhibitor treated cells. Intriguingly, treatment with VFA has been demonstrated to decrease the malignant phenotype in other cell lines [40]. Consequently, the identification of different patterns glycogene expression and lectin binding properties of HDAC inhibitor treated cells may play an important role in the development of innovative combinatorial approaches to cancer therapies.

Biological activity of the glycome is also dependent on the recognition of glycans by glycan binding proteins. SW13 cells exhibited binding to a broad number of glycan binding proteins in each of the eight classes present in the array. FK228 treatment significantly impacted the binding affinity for a number of glycans, but of particular interest was the 2-fold increase in α-Rha binding. Human serum contains an abundance of anti-carbohydrate antibodies, and anti-Rha antibodies are among the most abundant [41]. Further, rhamnose glycoconjugates have been demonstrated to target anti-carbohydrate antibodies specifically to tumor cells prompting rigorous investigations into their use for cancer immunotherapies, with very promising results [41–43]. Our findings that HDAC inhibitor treatment further increases the binding affinity for α-Rha raises the exciting possibility to use HDAC inhibitors as enhancers of cancer immunotherapy efficacy.

As mentioned above, HDAC inhibitors have largely proved ineffective as monotherapies for solid tumors, but have had some limited demonstrated efficacy in combination with other agents such as carboplatin and paclitaxel [44, 45]. In contrast, we observed decreased responsiveness to paclitaxel treatment following HDAC inhibition. It is worth noting that alterations in glycogene expression and cellular glycosylation patterns can significantly impact chemosensitivity [2, 4, 5]. Indeed, increased expression of ST8SIA4 in human leukemia and decreased expression of B4GALT2 in breast cancer cells has been associated with multidrug resistance [4, 5]. In SW13 cells, we noted both an increase in ST8SIA4 expression and a decrease in B4GALT2 expression in response to HDAC inhibition, which could help explain their decreased sensitivity to paclitaxel treatment. Findings from these studies underscore the importance of glycomic alterations in the progression of cancer and their utility in identification of effective chemotherapeutics. The utility of B4GALT2 and ST8SIA4 as prognostic indicators for HDAC inhibitor treatment effectiveness and chemotherapeutic sensitivity should be further investigated.

## Conclusions

HDAC inhibition substantially alters glycogene expression and significantly influences both glycan and glycan binding protein expression and most of these genes are related to oncogenesis. Importantly, the changes identified by the lectin and glycan arrays are consistent with a metastatic glycosignature and a more aggressive phenotype. Interaction between glycans and glycan binding proteins is key to the function of the dense layer of glycoconjugates on the cellular surface and both types of molecules are affected by HDAC inhibition. As such, we suggest that these changes in combination may result in substantial alterations oncogenic capacity observed in SW13 following after HDAC inhibitor treatment. The capacity for HDAC inhibition to mediate such widespread glycomic alterations in other cell lines remains to be determined. However, given the widespread use of HDAC inhibitors in cancer research, and yet their still limited utility as chemotherapeutic agents in solid tumors continued investigations are warranted.

## Additional file

Additional file 1: **Table S1.** **Table S2.** **Table S3.** **Table S4.** (ZIP 16600 kb)

## Abbreviations

5-Azd-dc: 5-Aza-2′-deoxycytidine
Gal: Galactose
GalNac: N-Acetylgalactosamine
Glc: Glucose
GlcNac: N-Acetylglucosamine
HDAC: Histone deacetylase
Neu: Neuraminic acid
Neu5Ac: N-Acetylneuraminic acid
Neu5Gc: N-Glycolylneuraminic acid
